# New real‐time PCR test for APOE genotyping in patients with Alzheimer’s disease before anti‐amyloid therapy

**DOI:** 10.1002/alz.087290

**Published:** 2025-01-09

**Authors:** Dirk Meißner, Annika Denker, Sonja Stallmann, Sarah Milbredt, Iswariya Venkataraman, Sandra Saschenbrecker, Ulf Steller

**Affiliations:** ^1^ Institute for Experimental Immunology, affiliated to EUROIMMUN Medizinische Labordiagnostika AG, Lübeck, Schleswig‐Holstein Germany; ^2^ EUROIMMUN US Inc, Mountain Lakes, NJ USA

## Abstract

**Background:**

In 2023, the FDA approved LEQEMBI, a monoclonal antibody therapy targeting beta‐amyloid plaques, for patients with early‐stage Alzheimer’s disease (AD). However, anti‐amyloid drug trials demonstrated an elevated risk for amyloid‐related imaging abnormalities with cerebral edema (ARIA‐E) in carriers of the AD risk allele apolipoprotein E epsilon 4 (APOE‐ɛ4), especially in ɛ4/ɛ4 homozygous individuals. Here, we report on the evaluation of a new real‐time polymerase chain reaction (PCR) test for APOE genotyping.

**Method:**

The EURORealTime APOE (EUROIMMUN), which enables simultaneous testing for all six APOE genotypes (ɛ2/ɛ2, ɛ2/ɛ3, ɛ2/ɛ4, ɛ3/ɛ3, ɛ3/ɛ4, and ɛ4/ɛ4) by real‐time PCR, was performed using genomic DNA (gDNA) isolated from EDTA blood samples. The PCR amplification results were evaluated using the EURORealTime Analysis Software (EUROIMMUN). Samples from healthy blood donors (HBD) were used to assess intra‐assay, inter‐assay and between‐lot precision (n=6) as well as analytical sensitivity (n=21), with each APOE genotype represented by at least one sample. Assay accuracy was analyzed by comparing genotype assignments to the results of the CE‐IVD‐marked EUROArray APOE (EUROIMMUN) and bidirectional Sanger sequencing using 110 samples (100 AD, 10 HBD). APOE genotype frequencies were compared between patients (100 AD) and controls (250 HBD).

**Result:**

Precision testing of the EURORealTime APOE resulted in 100% correct genotype calls. Assessment of the analytical sensitivity revealed valid and correct genotype assignment in all 21 samples, confirming 1 ng/µl as the recommended minimum gDNA concentration. The analytical accuracy amounted to 100% based on agreement with the two reference methods in 110/110 cases. Comparison of the APOE genotype distribution showed a higher proportion of ɛ4 allele carriers (i.e., ɛ2/ɛ4, ɛ3/ɛ4, and ɛ4/ɛ4) in AD patients than in HBD (50.0% vs. 25.2%). The prevalence of the ɛ4/ɛ4 genotype in these cohorts was 25.0% for AD patients compared with 2.8% for HBD.

**Conclusion:**

The well‐established high prevalence of the APOE‐ɛ4/ɛ4 genotype in AD patients, together with the increased risk of ARIA‐E in these homozygous carriers, substantiates the relevance of APOE genotyping prior to anti‐amyloid treatment. The EURORealTime APOE test provides sensitive and reliable determination of APOE alleles, thus presenting a suitable tool to improve risk stratification for potential LEQEMBI recipients.

